# Kava, Kawa, Kava-Kava—A Powerful Anæsthetic and Spinal Depressant

**Published:** 1886-12

**Authors:** 


ARTICLE IV.

### KAVA, KAWA, KAVA-KAVA—A POWERFUL ANÆSTHETIC AND SPINAL DEPRESSANT.

Since the advent of cocaine in its several forms for use as an anæsthetic ,the medical world has redoubled its efforts to find its equivalent, or better, its superior, and would seem to have met with encouragement, if not success, in the person of Dr. L. Lewin, of Berlin. We condense from the *Therapeutic Gazette* two of the leading contributions that have already appeared in regard to the substance and its effects on the system:

Kawa (Kawa-Kawa, Yakona, Yangona) is the root of Piper methysticum, a shrub three yards high, growing in Polynesia. The shrub is especially found on the New Hebrides, the Sandwich, Feejee, and Samoa Islands. The kawa-drinks reigned here until the introduction of alcohol, but to-day kawa is a favored drink in Polynesia, where kawa-houses exist like our cafes. Special men and women are selected to chew the root, then it is placed in vessels, water added, and stirred with the hands. The firm masses are then dissolved, and the kawa-drink is ready for use. The drink is served under prayers in half cocoa shells, and appears as a grayish dirty-looking fluid. The assumption that during the process of preparation a fermentation takes place is erroneous.

Chemically the root contains forty-nine per cent. of starch, numerous salts, and a non-nitrogenous body called kawahin. Lewin also found yangonin. Both these elements, however, have been proved to be inert. The actual active principle is a resinous mass of a fatty, aromatic taste, pungent and hot like pepper.

*Local Action.*—Kawa-resin increases the salivary secretion, and produces loss of sensitiveness immediately or shortly after application on all parts of the body. Taken into the mouth, one receives the sensation as if the part were burnt. Applied locally to the pharynx, the part grows anæsthetic even in the most sensitive persons. Gradually the increased salivary secretion and the anæsthesia of the pharynx disappear again. Applied to the conjunctiva of cold and warm-blooded animals, both conjunctiva and cornea lose the power of reaction to even the strongest irritation. The eyeball can be pressed, pinched, and rotated at will without producing a reaction. If the kawa-resin be injected into the connective tissue of an animal, anæsthesia ensues in the sphere surrounding the point of injection; mechanical and chemical irritation elicit no reflex response. At and surrounding the point of application no inflammatory symptoms appear, but, on the contrary, marked ischæmia.

*Constitutional Action.*—Ingested, the kawa-drink produces a sensation of comfort, peace and felicity, and never, as in the case of alcohol, desires of altercation or fight. In the mouth a peculiar. pleasant sensation of coolness is received, and may last for one or two hours. Consciousness and reason are in no way depressed, as in the case of opium, but the mental facilities are rather heightened. Fatigue and hardships appear easier borne under the influence of the drug. If larger quantities of the kawa-drink are ingested, a state of happy freedom of cares and a dreamy consciousness set in. The limbs grow feeble and powerless; gradually the will loses its control over the motor apparatus, rendering co-ordinate movements impossible. The person soon lies down, and gradually falls asleep, or rather in a condition of somnolency.

Nausea and headache, paresis of the extremities, and a nervous trembling are the usual sequelæ of kawa-ingestion after the somnolency has passed off. The system soon grows accustomed to the drug, and the kawa-habit is readily established.

*Animal Experiments.*—If kawa is administered to frogs per os or hypodermically, the animals soon grow feeble, the head sinks, and a state of motor incapacity ensues. Though voluntary motion has disappeared, reflex action is still active.

Lewin saw frogs in this condition for nine consecutive days; only the reaction to light and the slow cardiac action still testified to the life of the animals.

A general central paralysis is produced by kawa in a manner that the anterior horns of the gray substance, the motor ganglia, are first attacked, and later the sensible elements in the posterior horns of the gray substance, and lastly the brain ganglia. In birds, rabbits, cats, we obtain the same physiological effects. At first motion is apparently increased, the animals endeavor to run or fly away ; then this desire vanishes, and the animal lies down; temperature, respiration, and pulse fall gradually and simultaneously.

An alcoholic solution applied by the mouth or subcutaneously produces a deep sleep within a few minutes. Part of the kawa-resin is eliminated with the urine. The gastric mucous membrane shows, after death, an ischæmic appearance. These interesting observations clearly demonstrate that in kawa we have to deal with a drug of a most energetic activity, and that its power to produce complete local ischæmia and anæsthesia, reduction of the excitability of the spinal motor apparatus, and besides psychical quiescence, can be unquestionably utilized in therapeutics.

Kawa promises to become almost as valuable, provided it can be readily handled, as cocaine now is.

The resin is a semi-fluid body, with a peculiar aromatic taste, pungent and hot like pepper; when placed upon the tongue there is a momentary burning sensation, with increased salivary secretion, followed by local numbness, which may last for more than an hour. The kawa resin is too irritating to be applied to the human conjunctiva, but Dr. N. A. Randolph thinks that by first instilling cocaine, so as to render the conjunctiva insensitive, kawa might then be used to produce more prolonged anæsthesia. Its use in dental practice also suggests itself, and in the hands of aurists and laryngologists, though the facts that it is insoluble in water and glycerin, and that an alcoholic solution has serious disadvantages, will perhaps somewhat restrict its usefulness. It may also, perhaps, be used in suspension in mucilage.

Still another use for the resin of kawa or kâva, is in the treatment of gonorrhœa and acute affections of the urinary passages. The pain on micturition becomes less, the discharged decreases, and cure in many cases resulted in from ten to twenty days.—*Odontographic Journal.*

